# Efficacy and safety of repetitive transcranial magnetic therapy for post-stroke aphasia: a systematic review and meta-analysis of randomized controlled trials

**DOI:** 10.3389/fneur.2025.1614586

**Published:** 2025-10-20

**Authors:** Lin Xie, Yingxiu Diao, Cheng Gong, Jiahao Huang, Miao Huang, Zhenying Dong

**Affiliations:** ^1^Department of Rehabilitation Medicine, Ganzhou People’s Hospital, Ganzhou, Jiangxi, China; ^2^Department of Rehabilitation Medicine, Gannan Medical University, Ganzhou, Jiangxi, China; ^3^Department of Rehabilitation Medicine, Xiangya Hospital, Central South University, Jiangxi, China

**Keywords:** repetitive transcranial magnetic stimulation, post-stroke aphasia, systematic review, noninvasive brain stimulation, meta-analysis

## Abstract

**Objective:**

The purpose of this meta-analysis was to investigate the effectiveness and safety of repetitive transcranial magnetic stimulation (rTMS) in the treatment of patients with post-stroke aphasia (PSA).

**Methods:**

The PubMed, PEDro, Embase, Cochrane Library, CNKI, Wanfang Data and Web of Science databases were systematically searched from inception until January 30, 2024. Eligible randomized controlled trials (RCTs) contained information on the population (PSA), intervention (rTMS), and outcomes (Western Aphasia Battery, Aphasia Quotient, Aphasia Battery in Chinese, Boston Diagnostic Aphasia Examination, Aachener Aphasie Test, Concise Chinese Aphasia Test and Computerized Picture Naming Test). Participants in the rTMS intervention group were compared with those in sham or other control groups. Two independent researchers searched for, screened, and qualified the articles. Two independent researchers extracted key information from each eligible study. The authors’ names, year of publication, setting, total sample size, rTMS parameters, baseline/mean difference (MD), and 95% confidence interval (CI) were extracted using a standardized form, and the methodological quality was assessed using the Cochrane Risk of Bias tool (Revman 5.40, Nordic Cochrane Center) and GRADE (Grading of Recommendations, Assessment, Development, and Evaluation) system.

**Results:**

Thirty relevant RCTs were included, involving a total of 1,597 patients. The analysis turned out that rTMS combined with speech and language therapy (SLT) resulted in significant improvements in auditory comprehension, naming, repetition, and spontaneous speech in patients with PSA compared with sham stimulation combined with SLT or SLT alone in the control group. (auditory comprehension, MD = 1.94, 95%CI = [1.16, 2.17], *p* < 0.001; naming, MD = 1.53, 95%CI = [0.82, 2.24], *p* < 0.001; repetition, MD = 1.79, 95%CI = [1.20, 2.38], *p* < 0.001; spontaneous speech, MD = 1.97, 95%CI = [1.65, 2.29], *p* < 0.001).

**Conclusion:**

This meta-analysis showed that rTMS can safely and effectively promote the recovery of speech function in patients with PSA.

**Clinical trial registration:**

The study has been registered with Prospero https://www.crd.york.ac.uk/PROSPERO/search, (CRD42022363899).

## 1 Introduction

Post-stroke aphasia (PSA) refers to impaired or permanent loss of the ability to express and understand speech symbols caused by cerebrovascular disease, which results in a variety of language dysfunction, including listening, speaking, reading, and writing (1, 2). The incidence of PSA is high, with more than a third of stroke patients suffering from aphasia (3, 4). Due to the inability to communicate correctly, PSA patients are more prone to be depressed and anxious, which can seriously affect their quality of rehabilitation and life (5–7). Recent data show that stroke patients with aphasia incur significantly higher hospitalization costs for medical treatment, nursing, and related medical services than those without, which puts a huge burden on patients, their families, and society (8). The pathogenesis of PSA is not fully understood. However, some researchers have proposed the hypothesis that aphasia is related to the degree of lesions in the left hemisphere. When the lesions in the left hemisphere are small, the cortical area around the lesions in the ipsilateral hemisphere can play a role in compensating for ischemia. The right hemisphere’s corresponding speech-motor and language areas can functionally compensate for ischemia when the left hemisphere is extensively diseased (9, 10). Recently, researchers have also analyzed the mechanism of PSA from the perspective of neuroplasticity and explored possible intervention directions (11).

Common clinical treatment modalities for PSA include medication and speech training. Commonly used pharmacological treatments include dopaminergic, acetylcholinesterase inhibitors, and amino acid neurotransmitters (12). But of note, medication can only be used to improve some of the clinical symptoms of PSA patients. Due to the lack of uniform clinical standards for therapy, the effectiveness of speech and language training also varies from person to person (13–15). Early rehabilitation interventions include promoting communication outcome, functional restructuring, and blockade removal. Speech and language training (SLT) is highly recommended by the United State Stroke Foundation and the Australian Stroke Foundation as a significant treatment throughout aphasia (level 1A evidence) (15). In addition, the effectiveness of traditional SLT varies from person to person and may be due to a variety of factors, such as the patient’s level of aphasia, the technician’s individual nursing skills, communication strategies, and many other factors. Exploring novel, easy-to-implement, and efficient rehabilitation methods is still urgently needed to improve the clinical outcome of PSA further.

In recent years, with the development of non-invasive brain stimulation techniques, neuromodulation techniques such as transcranial direct current stimulation(tDCS) and repetitive transcranial magnetic stimulation (rTMS) have been widely used in the treatment of various clinical disorders, including depression (16), cognitive disorders (17), motor dysfunction (18), post-stroke dysphagia (19), PSA (20) and so on. Therefore, neuromodulation techniques are being used as novel therapeutic modalities that can complement the treatment of PSA. In addition, a recent net meta-analysis (21) showed that rTMS, a commonly used clinical neuromodulation technique, has better efficacy than tDCS in treating people with PSA. In most clinical settings, rTMS is rarely used as a stand-alone treatment for post-stroke aphasia. Instead, rTMS is typically administered in combination with speech and language therapy (SLT), which remains the gold standard rehabilitation approach. Some trials also paired rTMS with pharmacological treatments or cognitive training interventions. This concurrent use is based on the rationale that neuromodulation may enhance neuroplasticity, thereby amplifying the effects of behavioral therapies. Understanding these treatment pairings is essential for interpreting differences in efficacy across studies. This net meta-analysis demonstrated that therapeutic effects in the naming domain were moderated by the mean period of each therapy condition and the first language, while significant associations with age, therapy period, and number of sessions were observed for spontaneous speech. Overall, LF-rTMS is the most prioritized NIBS mode to alleviate global severity.

rTMS is a safe, painless, and easy-to-manipulate noninvasive neuromodulation technique that can modulate the excitability of cortical neurons on a temporal scale that exceeds the stimulation time course and on a spatial scale that exceeds the stimulation site (22). rTMS works by generating an induced magnetic field in order to induce secondary electrical currents in the adjacent neural tissues, which activate the cerebral cortex and changes the brain tissue-related physiological processes to achieve localization of cortical functions; At the same time, it can also improve local blood rheology and cortical metabolism by regulating the excitability of local brain tissues, affecting the release and transmission of neurotransmitters within the brain, and promoting the repair of damaged brain cells (22–24), and thus has been widely used in clinical rehabilitation.

Although previous review (20, 25) have discussed the therapeutic application of rTMS in patients with PSA, the clinical efficacy of rTMS in treating PSA patients, the optimal intervention parameters of rTMS and the safety of rTMS in the clinical treatment of PSA are still worthy of further analysis and exploration. And, recently, a number of new evidences of randomized controlled trials (RCTs) of rTMS for PSA have emerged. Thus, this meta-analysis aims to further explore the clinical efficacy and optimal intervention parameters of rTMS for PSA, and to provide a clinical evidence-based basis for the effective application of rTMS for PSA.

## 2 Methods

### 2.1 Protocol and registration

Our systematic review was designed and implemented based on the Preferred Reporting Items for Systematic Reviews and Meta-analysis (PRISMA) guideline (26). The study has been registered with Prospero (CRD42022363899).

### 2.2 Search strategy

In the initial screening, two researchers (CG and YXD) independently searched RCTs related to the topic in seven databases: Web of Science, PubMed, Embase, Cochrane Library, CNKI, Wanfang Data, and PEDro. Search for studies published between the date of database creation and September 28, 2022. We searched for standardized disease names in the International Classification of Diseases, 11th edition (ICD − 11). Ultimately, we identified the keywords for this study as “Stroke,” “Aphasia,” “Language Expression Disorder,” “Listening Comprehension Disorder,” and “Repetitive Transcranial Magnetic Stimulation.” In addition, we manually searched other relevant literature, such as studies included in some systematic reviews and meta-analyses, to broaden the search for eligible articles. As an example, the search strategy for the PubMed database is as follows (Table 1).

**Table 1:** The specific search strategy of PubMed database.

No. search items	
#1	Stroke [MESH]
#2	(Cerebrovascular Accident) OR (Brain Vascular Accident) OR (Cerebrovascular Accidents) OR (Strokes) OR (CVA) OR (CVAs) OR (Cerebrovascular Apoplexy) OR (Apoplexy, Cerebrovascular) OR (Vascular Accident, Brain) OR (Brain Vascular Accident) OR (Vascular Accidents, Brain) OR (Brain Vascular Accidents) OR (Cerebrovascular Stroke) OR (Cerebrovascular Strokes) OR (Stroke, Cerebrovascular) OR (Strokes, Cerebrovascular) OR (Apoplexy) OR (Cerebral Stroke) OR (Cerebral Strokes)
#3	#1 OR #2
#4	Aphasia [MESH]
#5	(Mixed Aphasia) OR (Global Aphasia) OR (Language Expression Disorder) OR (Listening Comprehension Disorder) OR (Motor Aphasia) OR (Broca Aphasia) OR (Wernicke Aphasia) OR (Alogia) OR (Alogia Acquired) OR (Aphasia) OR (Dysphasia)
#6	#4 OR #5
#7	Transcranial Magnetic Stimulation [MESH]
#8	(Repetitive Transcranial Magnetic Stimulation) OR(Magnetic Stimulation, Transcranial) OR (Magnetic Stimulations, Transcranial) OR (Stimulation, Transcranial Magnetic) OR (Stimulations, Transcranial Magnetic) OR (Transcranial Magnetic Stimulations) OR (Transcranial Magnetic Stimulation, Single Pulse) OR (Transcranial Magnetic Stimulation, Paired Pulse) OR (Transcranial Magnetic Stimulation, Repetitive) OR (TMS) OR (rTMS) OR (iTBS)
#9	#7 OR #8
#10	#3 AND #6 AND #9

### 2.3 Inclusion and exclusion criteria of the study

Included studies were required to follow our pre-defined inclusion and exclusion criteria strictly. According to the PICOS principles, the inclusion criteria of our review were as follows: (1) participants: patients diagnosed with post-stroke aphasia; (2) interventions: rTMS; (3) comparison: experimental group (rTMS) versus control group (placebo or no treatment) condition; (4) outcomes: Western Aphasia Battery (WAB), Aphasia Quotient (AQ), Aphasia Battery in Chinese (ABC), Boston Diagnostic Aphasia Examination (BADE), Aachener Aphasie Test (AAT), Concise Chinese Aphasia Test (CCAT) and Computerized Picture Naming Test (CPNT); (5) type of studies: RCT; (6) studies published in English or Chinese. Exclusion criteria for the literature: (1) duplicate data; (2) full-text content not available; (3) data not extractable.

### 2.4 Study selection

After completing the database search, we imported all retrieved studies into Endnote 20’s document management system (Endnote 20, United States) and removed duplicate studies using the software management function. Two researchers (CG and LX) then read the title and abstract of each study simultaneously and screened studies based on the inclusion and exclusion criteria we had previously developed. For initially screened studies, the two researchers would downloaded and read through the full text, removing articles that do not meet the inclusion criteria and discussed them to confirm their eligibility. If the two researchers disagree on the screening process of a study, the principal investigator (ZYD) was asked to provide advice and reach an agreement.

### 2.5 Data extraction

Two researchers (MH and LX) independently extracted the following data and items from the included literature: first author of the study, year of publication, the sample size of participating studies, age, gender, duration of disease, interventions tested, outcome indicators, and adverse effects. In addition, when the two researchers encountered difficulties in understanding or extracting the complete literature data during the data extraction process, the original authors of the literature would be contacted by sending an email to obtain the full trial data. When no response was received from the original author after three consecutive contacts, the study will be defined as missing data. Suppose two researchers disagree during the data extraction process. In that case, both would be placed in a research team with the Principal Investigator to discuss and resolve the issue. If the two researchers disagree on the screening process of a study, the principal investigator (ZYD) would be asked to provide advice and reach an agreement. The principal investigator will convene a meeting of the research team to discuss the reasons for any disagreements; once the sources of conflict are resolved, consensus will be reached.

### 2.6 Quality assessment

The quality assessment of the literature studies was completed independently by two researchers (MH and JHH), then discussed to produce consistent results. Risk bias was assessed using the Cochrane Risk of Bias tool (Revman 5.40, Nordic Cochrane Center). A total of seven items were considered: random sequence generation, allocation concealment, blinding of participants and personnel, blinding of outcome assessment, incomplete outcome data, selective reporting, and other biases. The risk bias assessment was mapped, and different colors differentiated the results into three levels: high risk—red, unknown risk—yellow and low risk—green. Heterogeneity between studies was statistically analyzed by Revman 5.40. The magnitude of heterogeneity was expressed as *I*^2^, with heterogeneity judged as high risk when *I*^2^ ≥ 75%, moderate risk when 75% > *I*^2^ ≥ 50%, low heterogeneity when 50% > *I*^2^ ≥ 25%, and no heterogeneity if *I*^2^ = 0% (27). I^2^ quantifies the extent of heterogeneity between studies. On the one hand, we select the appropriate effect model for the forest plot according to the magnitude of I^2^ to minimize the impact of high heterogeneity on the pooled results. On the other hand, during the assessment of evidence quality, we also use I^2^ to grade the strength of the evidence. The quality of evidence for outcome indicators was assessed using the Grading of Recommendations Assessment, Development, and Evaluation (GRADE) system, which examines study limitations, intermittency, inconsistency, and imprecision of results (28). The results were assessed by grading the evidence for the outcome indicators as “high,” “moderate,” “low,” or “very low,” and the strength of the recommendations was divided into two levels: “strong” and “weak” (29).

### 2.7 Statistical analysis

The extracted study data were entered into Revman 5.40 software^a^ for statistical and analytical purposes. The decision to use a fixed or a random effects model for the meta-analysis was based on the magnitude of heterogeneity. A random effects model was used when *I*^2^ ≥ 50%, and a fixed effects model was used when *I*^2^ < 50%. Mean difference (MD) and 95% confidence interval (CI) were used to express the effect size for studies using the same measure. Vice versa, standardized mean difference (SMD) and 95%CI were used to express the effect size for studies using different measures. *p* < 0.05, statistically significant.

### 2.8 Safety assessment

The number, type, and duration of adverse events that occurred during the rTMS intervention were counted in all the studies concerned. And statistically analyze the patients who experienced adverse events, as a percentage of the number of subjects. Record whether there were any intolerable or even life-threatening adverse reactions that caused the subjects to withdraw from the experiment. And, to track whether the adverse events in each study, still persisted during the follow-up time.

## 3 Results

### 3.1 Literature search findings

**Figure 1:**
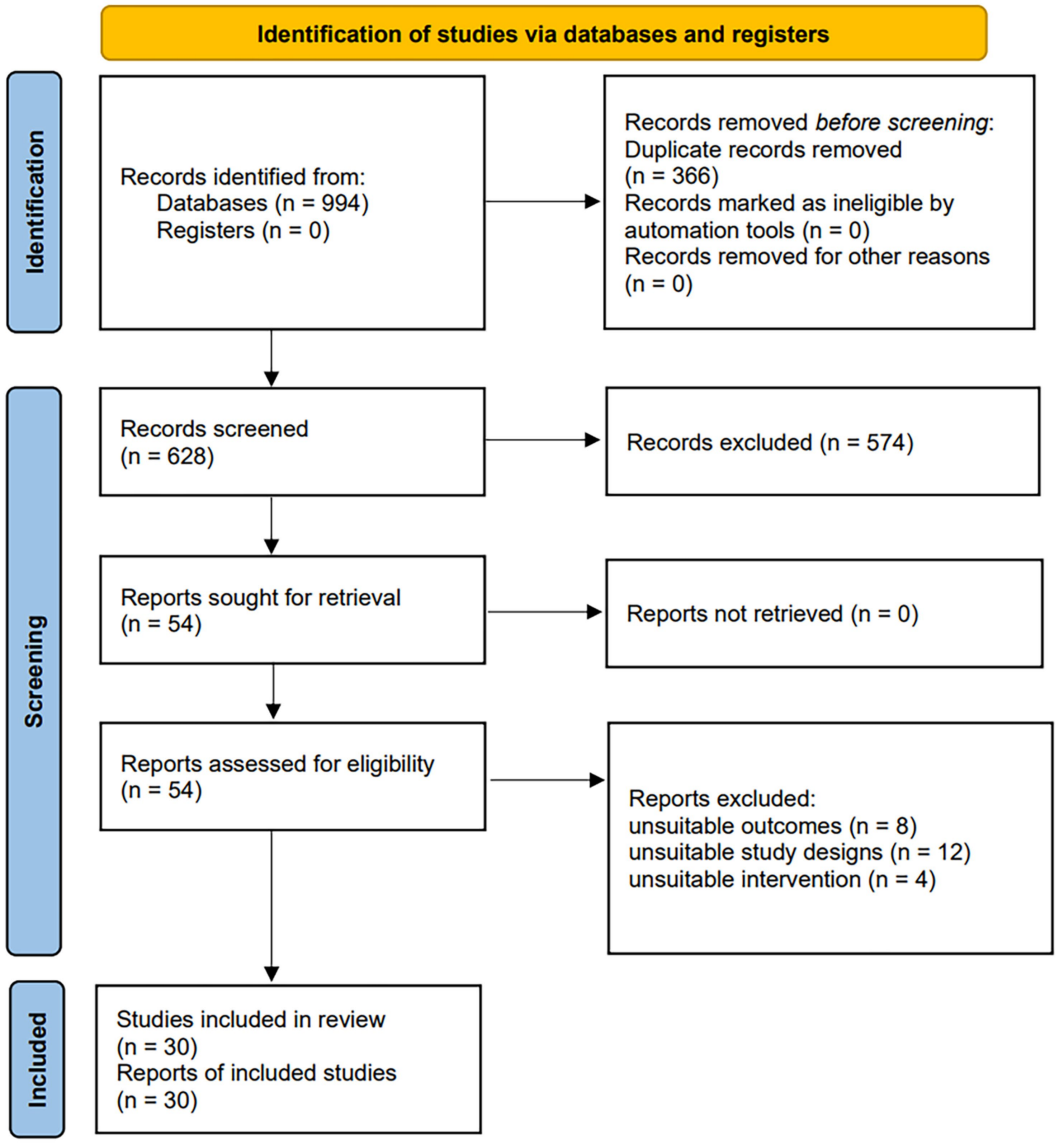
Flow graph of selection and exclusion.

A total of seven databases were searched for literature, and the initial search resulted in 994 studies. Duplicate studies were screened out and removed by software, leaving 628 studies. Two researchers (YZL and CG) read the titles and abstracts of these studies and screened out 574 that were irrelevant to the topic. The remaining 54 studies were downloaded in full and read through, and 24 studies were still excluded (8 studies were screened for not using the internationally accepted aphasia ratings listed in the inclusion criteria or data on outcome indicators were not fully available; 12 studies were screened for not strictly using a randomized controlled trial design; four studies were screened for not using rTMS as the primary intervention in an experimental comparison study). Finally, 30 eligible studies were included (30–59) (Figure 1).

### 3.2 Characteristics of included studies

Across the included randomized controlled trials, rTMS was most commonly applied in conjunction with SLT, whereas a smaller number of trials compared rTMS plus SLT with SLT alone or with sham stimulation plus SLT. Only a few studies combined rTMS with pharmacological agents. This variability in study design illustrates that rTMS is more appropriately viewed as an adjunctive rather than independent therapy for PSA. To improve clarity, we have minimized use of acronyms in the Results section, spelling out the assessment tools (e.g., Western Aphasia Battery instead of WAB on first mention). Table 2 summarizes the basic data of the 30 RCTs. A total of 1,597 patients with PSA were included, with sample sizes ranging from 12 to 120, of which 819 patients with PSA were treated with rTMS. Subjects’ aphasia types had non-fluent aphasia, Broca aphasia, Motor aphasia, Global aphasia, and Various aphasia. Among the included RCTs, the outcome indicators for rating aphasia in post-stroke patients included Western Aphasia Battery (WAB), Aachener Aphasia Test (AAT), Aphasia Battery in Chinese (ABC), Aphasia Quotient (AQ); Concise Chinese Aphasia Test (CCAT); Computerized Picture Naming Test (CPNT) and Boston Diagnostic Aphasia Examination (BDAE).

**Table 2:** The characteristic of the included studies.

Study	Gender (M/F)	Age (years)	Stroke duration	Aphasia type	Interventions	Outcome measures	Total time	Follow-up
1. Barwood et al. (59)	G1:2/4 G2: 1/5	G1: 60.8 ± 5.98 G2: 67 ± 13.11	3.49 ± 1.27 years 3.46 ± 1.53 years	Non-fluent aphasia	G1: SLT + rTMS G2: SLT + sham rTMS	BDAE	10 days	2 months
2. Chang (46)	G1;35/28 G2:33/30	67.3 ± 19.9 G2:66.4 ± 15.8	6.9 ± 3.1 days 7.3 ± 3.5 days	Broca aphasia	G1: SLT + rTMS G2: SLT + sham rTMS	WAB	15 days	No
3. Chen et al. (58)	G1;3/5 G2:3/4	65.7 66.5	<7 days	Broca aphasia	G1: SLT + rTMS G2: SLT + sham rTMS	ABC	10 days	2 weeks
4. Fan (48)	G1;25 G2:25	≥18	NR	NR	G1: SLT + rTMS G2: SLT + sham rTMS	ABC; AQ	20 days	No
5. Guo et al. (49)	G1:11/9 G2:12/8	62.1 ± 10.6 64.4 ± 8.5	33.1 ± 8.6 days 30.6 ± 9.4 days	Broca aphasia	G1: SLT + rTMS G2: SLT + sham rTMS	WAB; AQ	24 days	No
6. Haghighi et al. (47)	G1:3/3 G2:2/4	61.67 ± 7.06 60.50 ± 11.85	4–8 weeks	Broca aphasia	G1: SLT + rTMS G2: SLT + sham rTMS	WAB	10 days	No
7. Heiss et al. (55)	G1:15 G2:14	68.5 ± 8.19 69.0 ± 6.33	50.1 ± 23.96 days 39.7 ± 18.43 days	NR	G1: SLT + rTMS G2: SLT + sham rTMS	AAT	10 days	No
8. Hu et al. (4)	G1: 7/3 G2: 6/4 G3: 5/5 G4: 6/4	46.5 ± 12.1 48.5 ± 11.2 50.7 ± 10.4 47.3 ± 9.8	7.1 ± 2.7 months 7.5 ± 3.2 months 6.8 ± 2.3 months 7.7 ± 3.4 months	Non-fluent aphasic	G1: SLT + rTMS G1: SLT + rTMS G3: SLT + sham rTMS G4: SLT	WAB	2 weeks	2 months
9. Lai et al. (30)	G1:21/16 G2:20/17	62.01 ± 6.29 61.49 ± 6.36	1 ~ 3 months	Various	G1: SLT + rTMS G2: SLT + sham rTMS	AQ	8 months	No
10. Li et al. (43)	G1:9/6 G2:7/8	65.3 ± 5.6 68.3 ± 5.8	47.5 ± 7.4 days 51.0 ± 9.6 days	Motor aphasia	G1: SLT + rTMS G2: SLT + sham rTMS	AQ; WAB	3 weeks	3 weeks
11. Liu et al. (31)	G1:24/16 G2:26/14	54.1 ± 6.2 53.3 ± 5.4	58.4 ± 15.6 days 60.2 ± 14.3 days	NR	G1: SLT + rTMS G2: SLT + sham rTMS	AQ; WAB	4 weeks	No
12. Peng and Zhou (37)	G1:26/14 G2:27/13 G3:24/16	59.79 ± 5.58 59.80 ± 5.91 59.73 ± 5.82	10.4 ± 2.83 days 10.4 ± 2.76 days 10.37 ± 2.8 days	NR	G1: SLT + rTMS G2: SLT + sham rTMS G3: SLT	AQ; WAB	4 weeks	No
13. Qiu et al. (36)	G1:19/1 G2:18/2	55.00 ± 10.72 52.25 ± 15.00	2.12 ± 1.8 months 1.56 ± 1.6 months	Non-fluent aphasia	G1: SLT + rTMS G2: SLT + sham rTMS	WAB	4 weeks	No
14. Qu et al. (35)	G1:13/7 G2:14/6	68.60 ± 7.78 67.80 ± 7.32	26.5 ± 12.5 days 25.8 ± 11.8 days	Non-fluent aphasia	G1: SLT + rTMS G2: SLT + sham rTMS	AQ; WAB	2 weeks	No
15. Ren et al. (38)	G1:12/6 G2: 7/6 G3:9/6	65.95 ± 8.53 62.46 ± 10.95 63.60 ± 16.71	55.9 ± 19.4 days 50.6 ± 23.8 days 61.2 ± 22.7 days	Global aphasia	G1: SLT + rTMS G2: SLT + sham rTMS	WAB	3 weeks	No
16. Rubi-fessen et al. (50)	G1:5/10 G2:9/6	67.9 ± 8.12 69.6 ± 6.67	41.5 ± 21.5 days 48.7 ± 21.6 days	NR	G1: SLT + rTMS G2: SLT + sham rTMS	AAT	10 days	No
17. Seniów et al. (54)	G1:8/12 G2:10/10	61.8 ± 11.8 59.7 ± 10.7	33.5 ± 24.1 days 39.9 ± 28.9 days	Various	G1: SLT + rTMS G2: SLT + sham rTMS	BDAE	3 weeks	15 weeks
18. Shen (41)	G1:16/14 G2:17/13	57.31 ± 2.51 57.28 ± 2.35	3.75 ± 1.32 days 3.25 ± 1.25 days	NR	G1: SLT + rTMS G2: SLT	ABC	4 weeks	No
19. Tao (40)	G1:20/11 G2:18/13	60.2 ± 5.1 59.3 ± 4.5	NR	NR	G1: SLT + rTMS G2: SLT	AQ; ABC	4 weeks	No
20. Thiel et al. (53)	G1:13 G2:11	69.8 ± 7.96 71.2 ± 7.78	37.5 ± 18.5 days 50.6 ± 22.6 days	Various	G1: SLT + rTMS G2: SLT + sham rTMS	AAT	10 days	3 weeks
21. Tsai et al. (52)	G1:24/9 G2:17/6	62.3 ± 12.1 11.6 ± 4.3	17.8 ± 7.2 months 18.3 ± 8.2 months	Non-fluent aphasia	G1: SLT + rTMS G2: SLT + sham rTMS	CCAT	10 days	3 months
22. Waldowski et al. (56)	G1:6/7 G2:7/6	62.31 ± 11.03 60.15 ± 10.58	28.9 ± 19.4 days 48.5 ± 32.33 days	Various	G1: SLT + rTMS G2: SLT + sham rTMS	CPNT; BDAE	3 weeks	15 weeks
23. Wang et al. (51)	G1:14/1 G2:13/2	61.3 ± 13.2 60.4 ± 11.9	16.8 ± 6.4 months 16.1 ± 7.3 months	Non-fluent aphasia	G1: SLT + rTMS G2: SLT + sham rTMS	CCAT	2 weeks	3 months
24. Wang et al. (39)	G1:23/3 G2:11/4 G3:9/6	59.53 ± 1.37 57.00 ± 1.24 47.07 ± 1.37	< 3 months	NR	G1: SLT + rTMS G2: SLT + sham rTMS	WAB	2 weeks	No
25. Weiduschat et al. (57)	G1:1/5 G2:4/0	66.67 ± 8.26 63.75 ± 3.83	45.2 ± 21.0 days 57.5 ± 23.3 days	Various	G1: SLT + rTMS G2: SLT + sham rTMS	AAT	2 weeks	7 weeks
26. Fang et al. (45)	G1:28/20 G2:30/22	64.3 ± 15.7 63.5 ± 16.5	10.7 ± 3.5 days 10.7 ± 3.7 days	NR	G1: SLT + rTMS G2: SLT	AQ; WAB	4 weeks	No
27. Yang et al. (42)	G1:11/9 G2:10/10	46.34 ± 11.5 47.64 ± 13.6	6 months	NR	G1: SLT + rTMS G3: SLT	WAB	4 weeks	No
28. Yin et al. (33)	G1:24/26 G2:25/25	58.45 ± 3.50 57.35 ± 4.20	≤7 days	Various	G1: SLT + rTMS G3: SLT	AQ; ABC	4 weeks	No
29. Zhang et al. (34)	G1:30 G2:30	63.2 ± 10.3	NR	Motor aphasia	G1: SLT + rTMS G3: SLT	ABC	10 days	No
30. Zhou et al. (32)	G1:30/23 G2:28/25	61.25 ± 8.41 59.87 ± 7.64	9.35 ± 3.27 weeks 8.91 ± 2.36 weeks	Motor aphasia	G1: SLT + rTMS G3: SLT	AQ; WAB	4 weeks	No

M, male; F, Female; G1, group 1; G2, group 2; G3, group 3; G3, group4; SLT, speech and language training; rTMS, repetitive transcranial magnetic stimulation; NR, not report; AAT, Aachener Aphasie Test; CCAT, Concise Chinese Aphasia Test; CPNT, Computerized Picture Naming Test; AQ, Aphasia Quotient; ABC, Aphasia Battery in Chinese; WAB, Western Aphasia Battery; BADE, Boston Diagnostic Aphasia Examination.

In addition, Table 3 summarizes the intervention parameters of rTMS for post-stroke patients in each study, such as stimulation site, intensity, frequency, number of pulses, and stimulation time. Among the studies we included, 2 studies used rTMS at 0.5 Hz to treat PSA patients, 28 studies used rTMS at 1 Hz to treat PSA patients, and 3 studies used rTMS at 10 Hz to treat PSA patients. Overall, there are more clinical studies using low-frequency rTMS to treat PSA than high-frequency rTMS. And low-frequency rTMS treatment is mainly 1 Hz rTMS.

**Table 3:** Main parameters of rTMS.

Study	Parameters					Adverse events and rates
	Frequency	Stimulation location	Intensity	Number of pulses a day	Stimulation time	
1. Barwood et al. (59)	1 Hz	The anterior portion of homolog to right pars triangularis in Broca’s area	90% RTM	1,200 pulses	20 min a day, 10 days	No
2. Chang (46)	1 Hz	Broca’s area in the right hemisphere	80% RTM	500 pulses	20 min a day, 15 days	No
3. Chen et al. (58)	1 Hz	Broca’s area in the right hemisphere	80% RTM	500 pulses	20 min a day, 5 days a week, 2 weeks	No
4. Fan (48)	1 Hz	No report	90% RTM	1,200 pulses	20 min a day, 5 days a week, 4 weeks	No
5. Guo et al. (49)	1 Hz	Right side hemispheric language mirror area	70%RTM	1,800 pulses	30 min a day, 6 days a week, 4 weeks	Headache; nausea <24 h (*n* = 2/40)
6. Haghighi et al. (47)	1 Hz	The inferior posterior frontal gyrus	100%RTM	No report	20 min a day, 5 days a week, 2 weeks	No
7. Heiss et al. (55)	1 Hz	Contralesional inferior frontal gyrus	90%RTM	No report	20 min a day, 5 days a week, 2 weeks	No
8. Hu et al. (44)	G1: 1 Hz G2: 10 Hz	Mirror area within Broca’s area	80%RTM	600 pulses	10 min a day, 5 days a week, 2 weeks	Dizziness <24 h (*n* = 1/20)
9. Lai et al. (30)	1 Hz	Broca or Wernicke area in the right hemisphere	90%RTM	1,200 pulses	20 min a day, 5 days a week, 8 weeks	No
10. Li et al. (43)	1 Hz	Broca’s mirror area in the right hemisphere	80%RTM	1,200 pulses	20 min a day, 5 days a week, 3 weeks	No
11. Liu et al. (31)	10 Hz	Broca’s and Wernicke’s zones in the left hemisphere	90%RTM	1,200 pulses	10 min a day, 5 days a week, 4 weeks	No
12. Peng and Zhou (37)	1 Hz	Broca’s area in the right hemisphere	80%RTM	960 pulses	20 min a day, 5 days a week, 4 weeks	No
13. Qiu et al. (36)	1 Hz	Broca’s mirror area in the right hemisphere	80%RTM	1,200 pulses	once a day, 5 days a week, 4 weeks	Dizziness <24 h (*n* = 1/20)
14. Qu et al. (35)	1 Hz	Broca’s area in the right hemisphere	100% RTM	1,200 pulses	once a day, 5 days a week, 2 weeks	No
15. Ren et al. (38)	1 Hz	G1: The homolog of the left Broca’s area; G2: The homolog of the left Wernicke’s area	80%RTM	1,200 pulses	20 min a day, 5 days a week, 3 weeks	No
16. Rubi-fessen et al. (50)	1 Hz	The right triangular part of the inferior frontal gyrus	90%RTM	No report	20 min a day, 5 days a week, 2 weeks	No
17. Seniów et al. (54)	1 Hz	The right-hemisphere homolog of Broca’s area	90%RTM	1800 pulses	20 min a day, 5 days a week, 3 weeks	No
18. Shen (41)	0.5 Hz	Language mirror area of the cerebral hemisphere	80%RTM	600 pulses	22 min a day, 5 days a week, 4 weeks	No
19. Tao (40)	1 Hz	No report	No report	1,200 pulses	23 min a day, 7 days a week, 4 weeks	No
20. Thiel et al. (53)	1 Hz	The right triangular part of the posterior inferior frontal gyrus	90%RTM	No report	20 min a day, 5 days a week, 2 weeks	No
21. Tsai et al. (52)	1 Hz	The contralesional pars triangularis	90%RTM	600 pulses	10 min a day, 5 days a week, 2 weeks	No
22. Waldowski et al. (56)	1 Hz	Two parts of Broca’s area homologs: the anterior part and posterior part	90%RTM	No report	30 min a day, 5 days a week, 3 weeks	No
23. Wang et al. (51)	1 Hz	The contralesional target area	90%RTM	1,200 pulses	20 min a day, 5 days a week, 2 weeks	No
24. Wang et al. (39)	G1: 1 Hz G2: 0.5 Hz	Broca’s area of the left cerebral hemisphere	90%RTM	1,200 pulses	20 min a day, 5 days a week, 2 weeks 40 min a day, 5 days a week, 2 weeks	No
25. Weiduschat et al. (57)	1 Hz	The right triangular part of the inferior frontal gyrus	90%RTM	No report	20 min a day, 5 days a week, 2 weeks	No
26. Fang et al. (45)	G1: 10 Hz G2:1 Hz	G1: Broca’s area of the left cerebral hemisphere G2: Broca’s area of the right cerebral hemisphere	80%RTM	1,000 pulses	20 min a day, 5 days a week,4 weeks	No
27. Yang et al. (42)	1 Hz	Right inferior frontal gyrus triangle	80%RTM	480 pulses	20 min a day, 5 days a week,4 weeks	No
28. Yin et al. (33)	1 Hz	Broca’s and Wernicke’s zones in the right hemisphere	40% ~ 90%RTM	800 pulses	20 min a day, 5 days a week,2 weeks	No
29. Zhang et al. (34)	1 Hz	Broca’s area of the right cerebral hemisphere	80%RTM	500 pulses	30 min a day, 10 consecutive days	No
30. Zhou et al. (32)	1 Hz	Broca’s area of the right cerebral hemisphere	90%RTM	1,200 pulses	20 min a day, 5 days a week, 4 weeks	No

### 3.3 Quality assessment result

**Figure 2:**
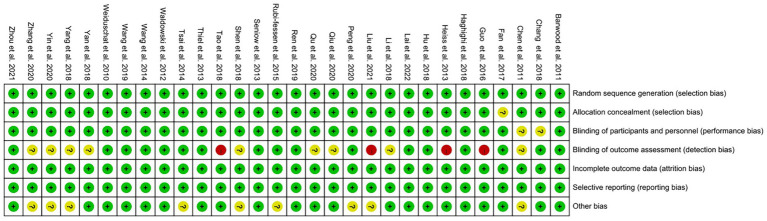
Risk of bias summary of included studies.

**Figure 3:**
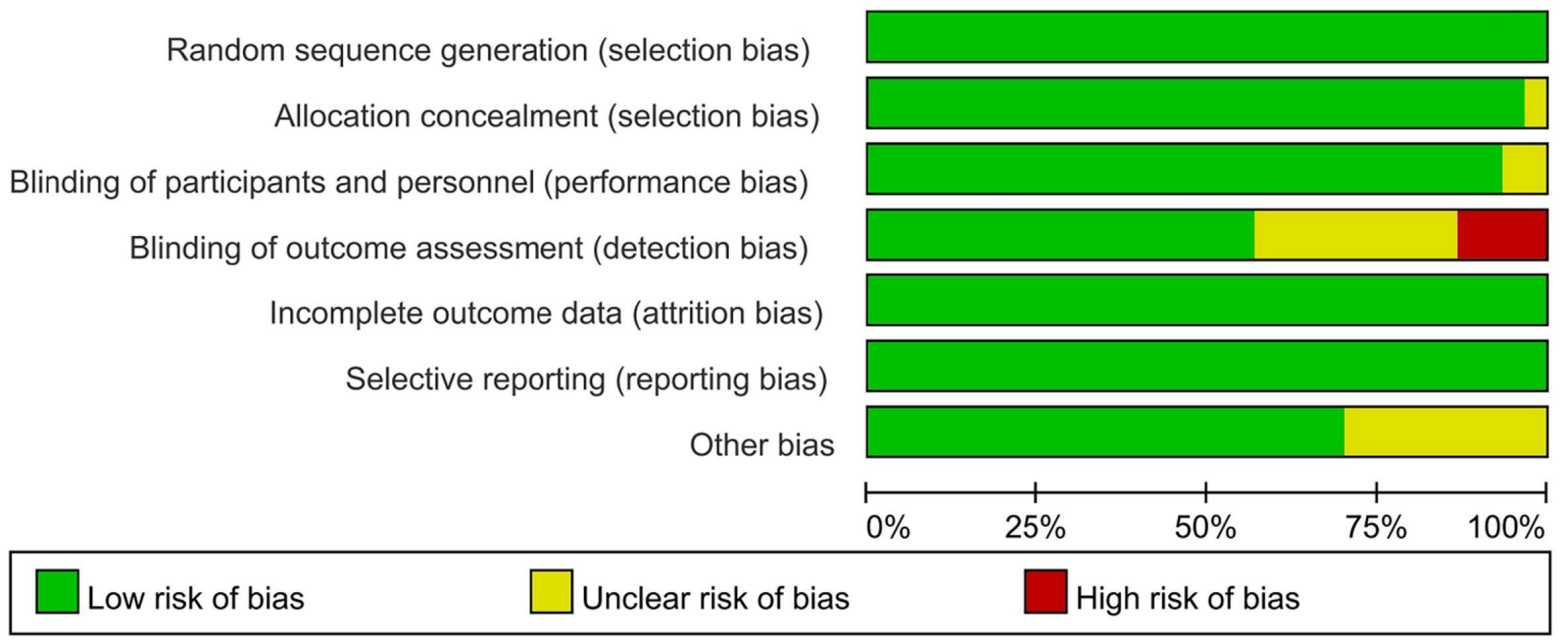
Risk of bias graph of included studies.

The risk of bias assessment showed that in all included RCTs, four RCTs (31, 40, 49, 55) did not use blinding for the assessment of outcome indicators and had a high risk of detection bias. And nine studies (31, 33, 34, 37, 41, 45, 50, 52, 58) did not explicitly report blinding for assessing outcome indicators, and the risk of detection bias was unclear. The risk of bias was low for all RCTs in the other items evaluated for risk of bias. Overall, the risk of bias was low in our included studies (Figures 2, 3).

We evaluated the level of evidence for the outcome indicators of the included studies by GRADE. Three outcome indicators were rated as intermediate, (AQ, ABC and WAB) due to high heterogeneity between studies (*I* ^2^ > 80%) and were therefore downgraded in the inconsistency assessment. Four outcome indicators (AAT, BADE, CCAT and CPNT) were rated as intermediate because the sample sizes were too small (*n* < 100), which tended to influence the imprecision of the study results, and were downgraded in the imprecision assessment. The remaining outcome indicators were not found to be downgraded factors in each of the GRADE assessments. Overall, the GRADE recommended evidence level for the outcome indicator was “strong” (Table 4).

**Table 4:** Grading of recommendations assessment, development, and evaluation (GRADE) quality of evidence.

Assessment content	Outcomes						
	AAT	ABC	AQ	BADE	CCAT	CPNT	WAB
Number of studies	4	7	16	2	2	3	12
Design	RCT	RCT	RCT	RCT	RCT	RCT	RCT
Study limitations	0	0	0	0	0	0	0
Inconsistency	0	−1*	−1*	0	0	0	−1*
Indirectness	0	0	0	0	0	0	0
Imprecision	−1^#^	0	0	−1^#^	−1^#^	−1^#^	0
Publication bias	0	0	0	0	0	0	0
Effect size	0	0	0	0	0	0	0
GRADE quality	Moderate	Moderate	Moderate	Moderate	Moderate	Moderate	Moderate
Symbolic expression	⊕ ⊕ ⊕⊖	⊕ ⊕ ⊕⊖	⊕ ⊕ ⊕⊖	⊕ ⊕ ⊕⊖	⊕ ⊕ ⊕⊖	⊕ ⊕ ⊕⊖	⊕ ⊕ ⊕⊖

AAT, Aachener Aphasie Test; ABC, Aphasia Battery in Chinese; AQ, Aphasia Quotient; BADE, Boston Diagnostic Aphasia Examination. CCAT, Concise Chinese Aphasia Test; CPNT, Computerized Picture Naming Test; WAB, Western Aphasia Battery. *High heterogeneity (I^2^ > 80%); ^#^the sample size was too small (*n* < 100).

### 3.4 Results of statistical analysis

**Figure 4:**
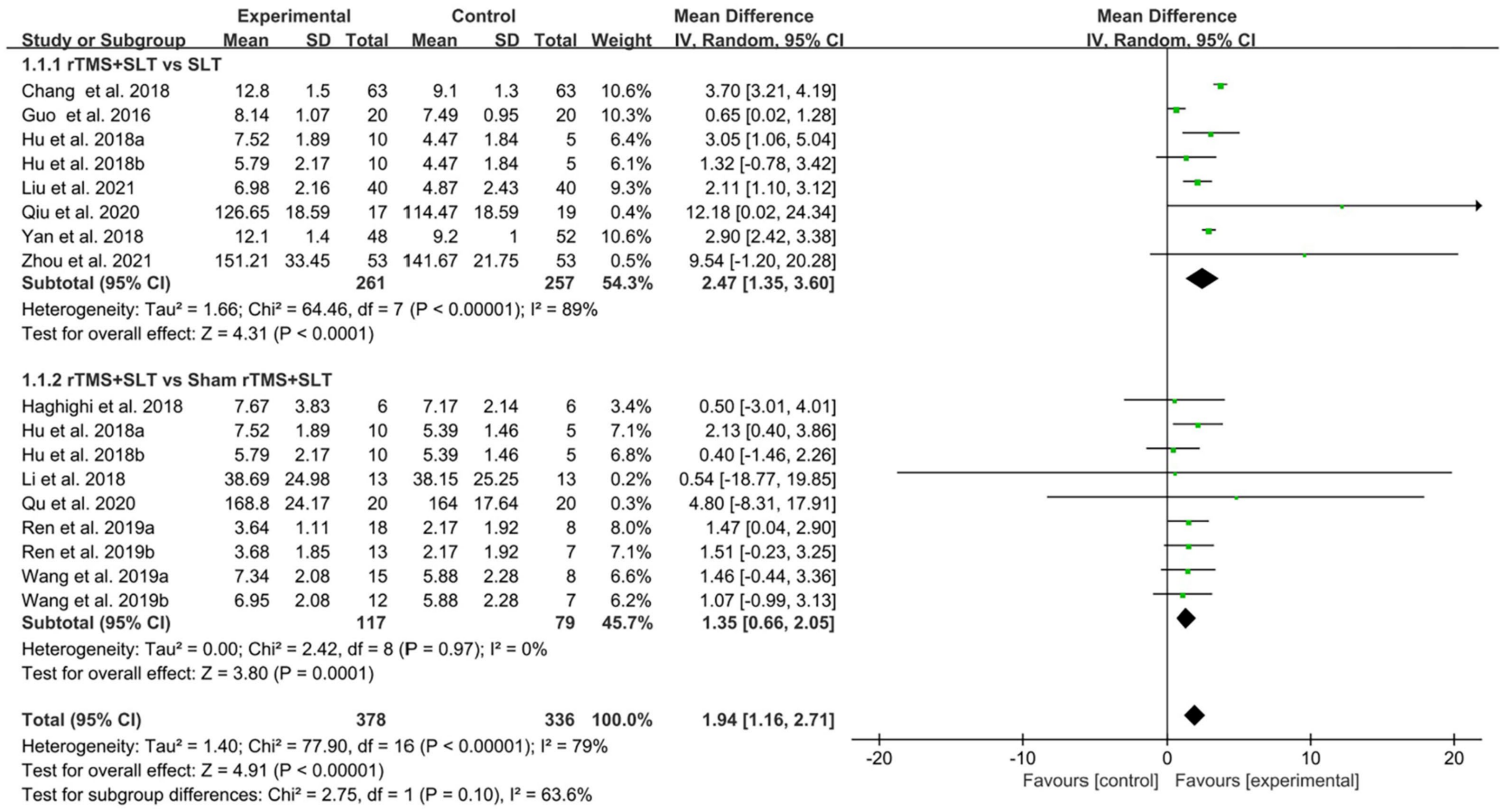
Forest plot for auditory comprehension (Western Aphasia Battery).

**Figure 5:**
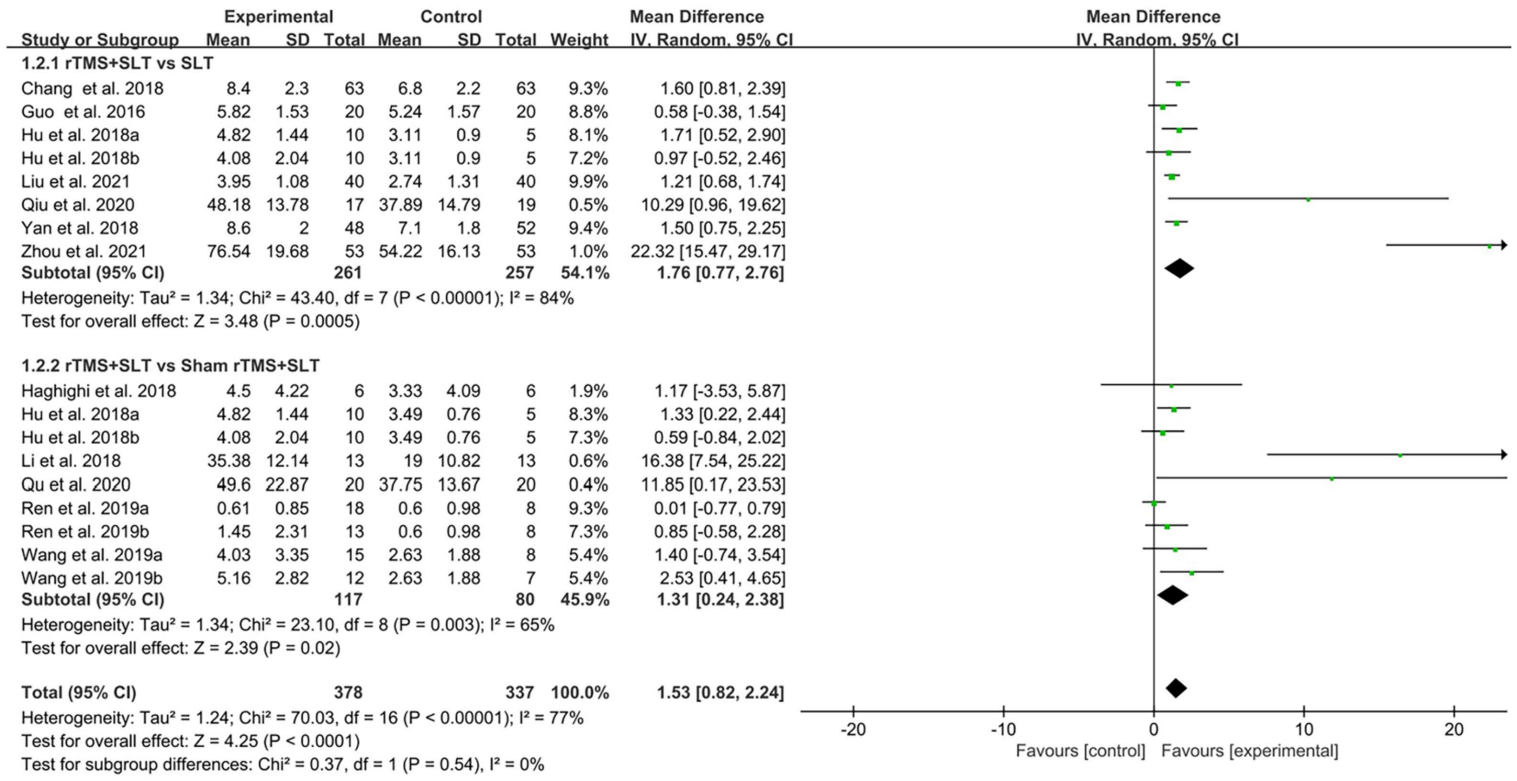
Forest plot for naming (Western Aphasia Battery).

**Figure 6:**
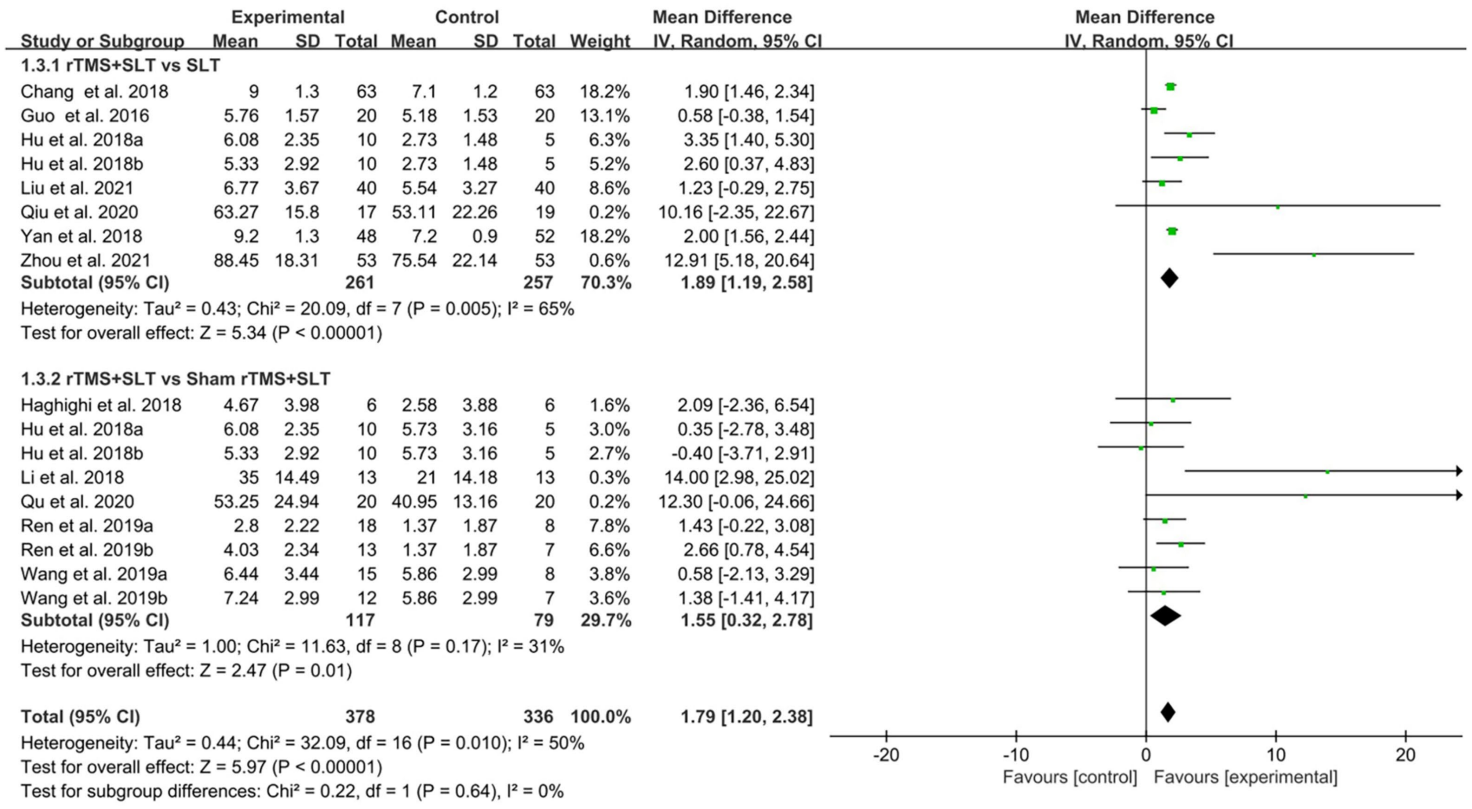
Forest plot for repetition (Western Aphasia Battery).

**Figure 7:**
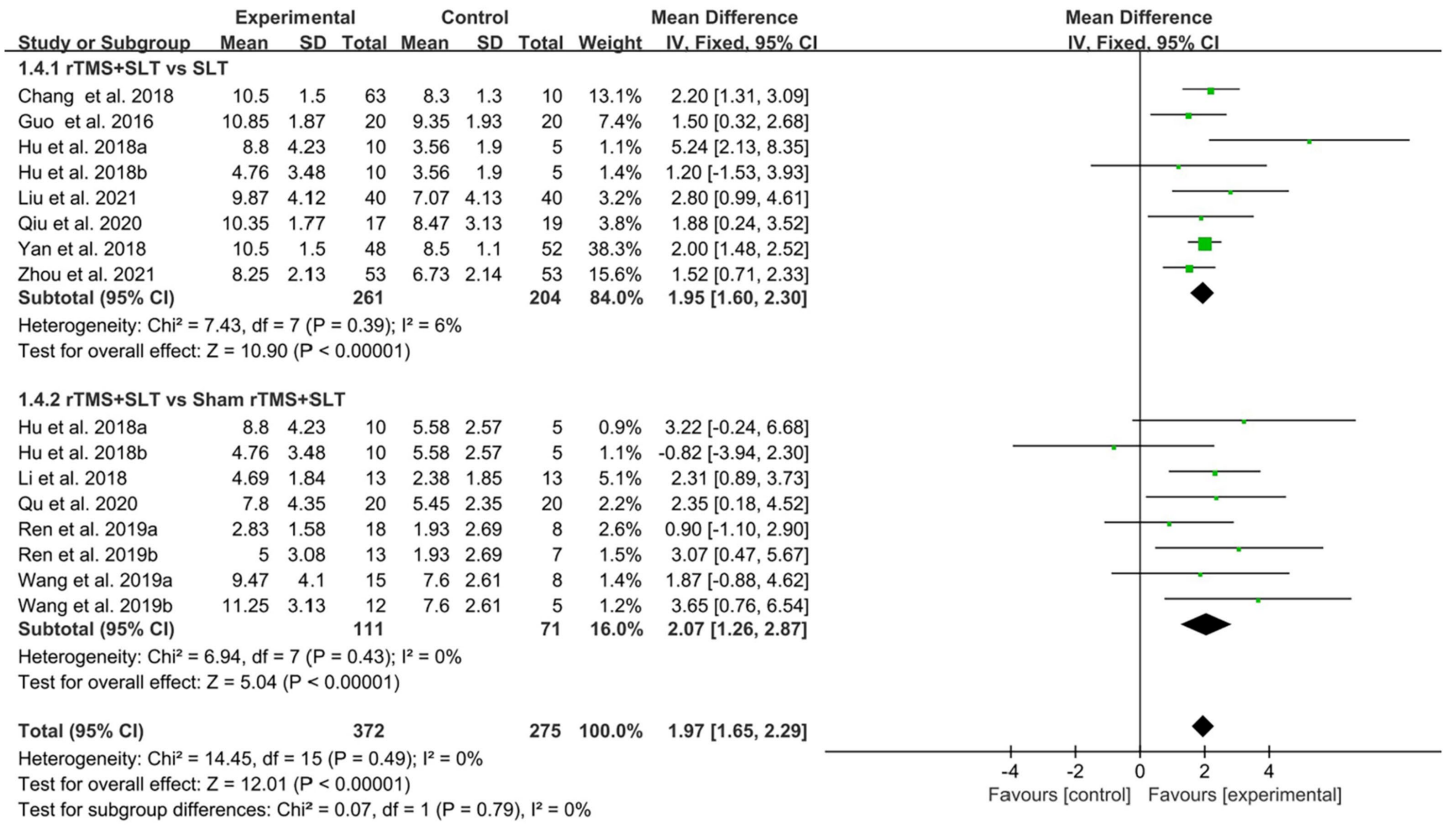
Forest plot for spontaneous speech (Western Aphasia Battery).

There are 12 studies (31, 32, 35, 36, 38, 39, 43–47, 49) rated the speech function of PSA patients by WAB with *I*^2^ > 50% between studies and therefore used a random-effects model for data analysis. The results of the forest plot analysis showed that patients treated with rTMS had greater improvements in areas of verbal comprehension and expression. The specific improvement results were as follows: rTMS was more effective in improving auditory comprehension in PSA patients compared to control group (MD = 1.94, 95% CI = [1.16, 2.17], *I*^2^ = 79%, *p* < 0. 001, Figure 4); rTMS was more effective in improving naming ability in PSA patients compared to control group (MD = 1.53, 95% CI = [0.82, 2.24], *I*^2^ = 77%, *p* < 0. 001, Figure 5); rTMS was more effective in improving verbal repetition in PSA patients compared with the control group (MD = 1.79, 95% CI = [1.20, 2.38], *I*^2^ = 50%, *p* < 0.001, Figure 6); and rTMS was more effective in improving PSA patients’ spontaneous speech (MD = 1.97, 95% CI = [1.65, 2.29], *I*^2^ = 0%, *p* < 0.001, Figure 7).

**Figure 8:**
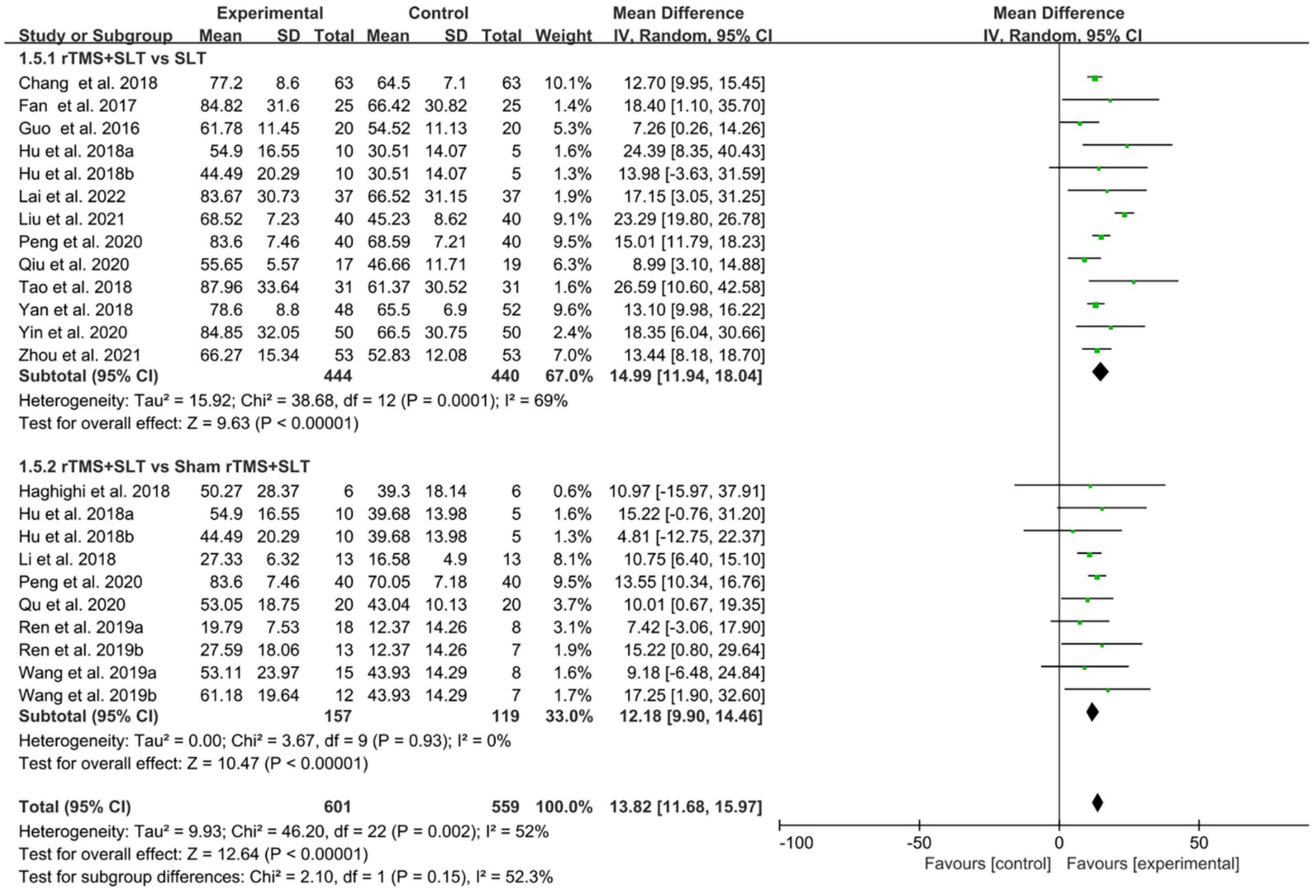
Forest plot for Aphasia Quotient (AQ).

The degree of impairment in PSA patients was assessed in 16 studies (30, 32, 33, 35–40, 43–49) using AQ scores with an *I*^2^ > 50% between studies, so the data were analyzed using a random effects model. The results of data analysis showed that compared to the control group, the experimental group showed better improvement in AQ scores than the control group (MD = 13.82, 95% CI = [11.68, 15.97], *I*^2^ = 52%, *p* < 0.001; Figure 8).

**Figure 9:**
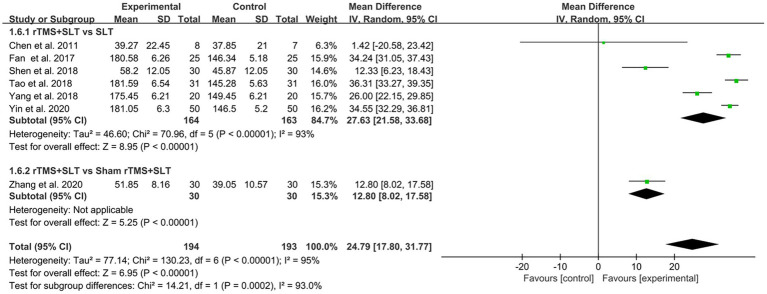
Forest plot for Aphasia Battery in Chinese (ABC).

The degree of language loss in PSA patients was assessed by ABC in 7 studies (33, 34, 40–42, 48, 58) with *I*^2^ > 50% between studies, and we analyzed the data using a random-effects model. The results of the forest plot analysis showed that compared to the control group, the experimental group had better outcomes in ABC scores (MD = 24.79, 95% CI = [17.80, 31.77], *I*^2^ = 95%, *p* < 0.001; Figure 9).

**Figure 10:**
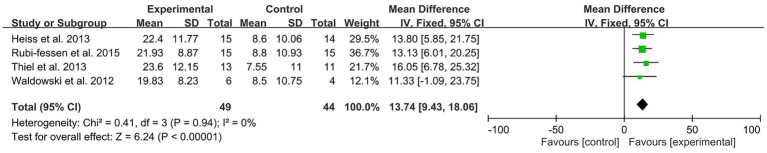
Forest plot for Aachener Aphasie Test (AAT).

In 4 studies (50, 53, 55, 56), the verbal function of PSA patients was assessed using the AAT scale, with an *I*^2^ < 50% between studies, and we analyzed the data using a fixed effects model. The results of the forest plot analysis showed that compared to the control group, the experimental group showed a significant improvement in AAT scores compared with the control group (MD = 13.74, 95% CI = [9.43, 18.06], *I*^2^ = 0%, *p* < 0.001; Figure 10).

**Figure 11:**
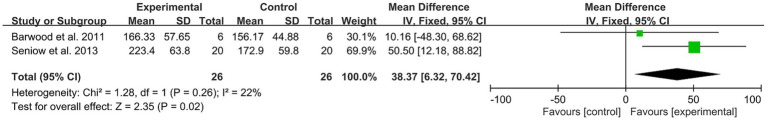
Forest plot for Boston Diagnostic Aphasia Examination (BADE).

The severity of aphasia in PSA patients was assessed in two studies (54, 59) using the BADE scale, with an *I*^2^ < 50% between studies, and we analyzed the data using a fixed effects model. The results of the analysis of the forest plot showed that the experimental group had a better improvement than the control group in terms of BADE scores in patients with PSA (MD = 38.37, 95% CI = [6.32, 70.42], *I*^2^ = 22%, *p* = 0.02; Figure 11).

**Figure 12:**
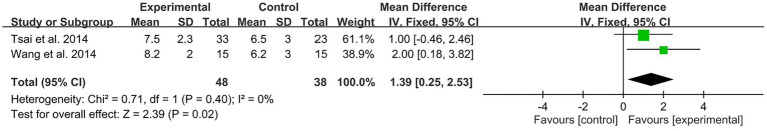
Forest plot for Concise Chinese Aphasia Test (CCAT).

Two studies (51, 52) used the CCAT scale to assess language function in PSA patients, and the *I*^2^ value between studies was 0%, thus the data were analyzed using a fixed effects model. The results of the analysis of the forest plot showed that the experimental group had a more positive contribution in improving the CCAT scores of PSA patients compared to the control group (MD = 1.39, 95% CI = [0.25, 2.53], *I*^2^ = 0%, *p* = 0.02; Figure 12).

**Figure 13:**
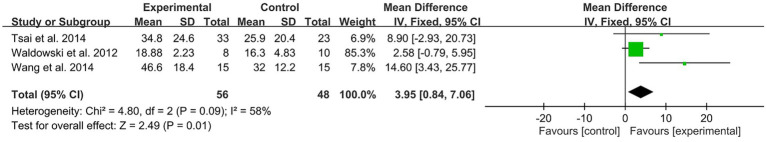
Forest plot for Computerized Picture Naming Test (CPNT).

In addition, 3 studies (51, 52, 56) tested the naming function of PSA patients by CPNT alone, with an *I*^2^ < 50% between studies, so the data were analyzed using a fixed effects model. The results of the forest plot analysis showed that the experimental group was more able to improve the naming ability of PSA patients and promote the recovery of verbal function compared to the control group (MD = 3.95, 95% CI = [0.84, 7.06], *I*^2^ = 8%, *p* = 0.01; Figure 13).

### 3.5 Adverse event reporting results

Of the 30 studies we included, only three studies reported the occurrence of adverse events. One of these studies (49) reported that two participants treated with rTMS experienced transient headache and nausea with a duration of <24 h, which accounted for 2/819 of the total number of participants in the experimental group of our study. Two other studies (36, 44) reported transient dizziness in two subjects treated with rTMS for <24 h, which accounted for 2/819 of the total number of participants in the experimental group of our study. In summary the number of patients with PSA treated with rTMS who developed adverse events as a proportion of the total number of participants in the experimental group was 4/819. In addition, based on the results reported in all studies, no patients withdrew from the experimental studies due to exhibited excessive adverse reactions. Moreover, only three of the 30 included studies reported adverse events (reporting rate 10%), and all RCTs had small sample sizes (*n* < 100). Therefore, we must consider the possibility of publication bias arising from unrecorded or unreported adverse events, which could underestimate the true risks of rTMS and thereby overstate its safety. We recommend that future studies continue to adhere strictly to established rTMS safety guidelines to ensure rigorous practice.

## 4 Discussion

The aim of this meta-analysis was to determine the efficacy of rTMS on the rehabilitation of speech function in patients with PSA. In the analysis obtained so far, we found that rTMS can effectively promote the recovery of speech function in PSA patients, which is consistent with the partial results of previous studies (60–62). Previous studies used rTMS as the intervention in PSA patients and employed speech-function scales (WAB, AQ, ABC, etc.) as outcome measures; they likewise demonstrated that rTMS can effectively improve language abilities in this population, but none assessed the safety of rTMS for PSA. The analysis with WAB as the assessment outcome showed that rTMS combined with SLT treatment was more effective than SLT alone in treating patients with PSA, as evidenced by the improvement in patients’ language abilities such as auditory comprehension, naming, repetition, and spontaneous speech. Compared with previous meta-analyses (20, 25), we have not only added recently published RCTs but also widened the spectrum of stimulation frequencies employed across studies and incorporated a broader array of outcome measures to provide more comprehensive assessments. Meanwhile, the improvement of the results assessed by CCAT and AAT indicated that rTMS could effectively enhance the speech function of PSA patients. In addition, the improvement of the assessment results by AQ, ABC and BADE showed that rTMS could effectively reduce the degree of aphasia impairment in PSA patients. In addition, to provide a higher level of evidence support, we conducted a more in-depth analysis and discussion of the mechanism of action of rTMS in treating PSA patients and the treatment effects of different intervention parameters.

Currently, more researchers prefer the “hemispheric balance theory” for the treatment rationale of rTMS in stroke patients (63, 64). An important factor influencing treatment efficacy is the site of stimulation. In the majority of trials, rTMS was delivered to the contralesional hemisphere, most often the right inferior frontal gyrus or its homolog of Broca’s area. Several studies, however, applied stimulation to lesioned hemisphere regions or adopted bilateral protocols. While our data were not sufficient to conduct subgroup meta-analysis of stimulation site, existing evidence suggests that site-specific modulation may differentially affect language outcomes in patients with Broca-type, global, or motor aphasia. Likewise, pairing rTMS with behavioral interventions such as SLT appears to maximize recovery potential compared to rTMS alone. Future large-scale trials should stratify patients according to stimulation target and aphasia profile to clarify whether specific protocols yield superior outcomes. In our brain, the bilateral hemispheres are in a state of equilibrium of mutual inhibition under normal physiological conditions, usually called “transcallosal mutual inhibition.” However, the hemispheric equilibrium of mutual inhibition can be disrupted in stroke patients with brain damage. For example, motor aphasia occurs in patients with damage to the Broca’s area in the left hemisphere, resulting in a decrease in the inhibitory capacity of the right hemisphere, which in turn leads to an activation of the right hemisphere and an increase in the inhibitory effect of the right hemisphere on the left hemisphere, thus breaking the balance of bilateral hemispheric inhibition and affecting the recovery of speech function in patients with post-stroke aphasia (63, 64). In order to correct the imbalance between the two hemispheres, we need to regulate the excitability of both cortices, and rTMS can do just this. It has been shown that rTMS can produce an electric field in the brain based on the principle of electromagnetic induction, which induces depolarized neurons to regulate cortical excitability (65). It has been found that high-frequency (>1 Hz) rTMS increases cortical excitability and low-frequency (≤1 Hz) rTMS decreases cortical excitability (66–68). and through this mechanism, rTMS can regulate the imbalance in both hemispheres (69–71) and cause plasticity changes in the cerebral cortex, thus promoting the recovery of speech function in post-stroke aphasic patients (50, 72). Thiel et al. (53) investigated the mechanism of rTMS using fMRI and found that rTMS could inhibit the hyperactivation of the healthy hemisphere, which led to a decrease in the inhibitory ability of the healthy hemisphere on the language control area of the affected hemisphere and promoted the rebalancing of the bilateral hemispheres, thus improving the language function of patients with post-stroke aphasia.

Among the 30 RCTs included, only three employed high-frequency rTMS; the remainder used low-frequency stimulation, and no uniform outcome measures were adopted. Thus, the available data are insufficient for a subgroup analysis comparing the efficacy of high- versus low-frequency rTMS. And the results of forest plot data show that both high-frequency rTMS and low-frequency rTMS can have a positive therapeutic effect on aphasia in stroke patients. Combined with the balance theory of both human hemispheres (73), there are good reasons to try the combination of high-frequency rTMS and low-frequency rTMS and to conduct a comparative efficacy study with low-frequency rTMS or high-frequency rTMS alone to explore the best treatment option of rTMS for post-stroke aphasia treatment. Yan et al. (45) reported in the previous study that combining high-frequency rTMS with low-frequency rTMS can effectively promote the recovery of speech function in stroke patients. Moreover, Hu et al. (44) also proven that low-frequency rTMS had superior and longer-lasting therapeutic effects than high-frequency rTMS on the recovery of speech function in patients with non-fluent aphasia, especially in the areas of spontaneous speech, aphasia quotient, and auditory comprehension function. However, a study by Wang et al. (39) showed that there was no difference in the therapeutic effect of low-frequency rTMS of different frequencies on patients with PSA. It can be seen that more, multicenter, RCTs with large sample sizes of high-frequency rTMS in combination with low-frequency rTMS are still needed to further approach the optimal intervention parameters of rTMS for post-stroke aphasia in the future.

Wang et al. (39) and Shen (41) expanded the selection of parameters of the commonly used rTMS and conducted a comparative study of the efficacy of 0.5-Hz rTMS and 1-Hz rTMS on PSA patients. The results found that patients in the sham stimulation group, both 0.5 Hz group, and 1 Hz group all had better WAB scores after treatment. Moreover, there was no statistically significant difference in the efficacy between the 0.5 Hz group and the 1 Hz group in treating patients with PSA, nor was there a significant difference in the improvement of WAB scores in PSA patients. However, the two groups were not identical regarding improvement in speech function. With the extension of treatment time, the 0.5 Hz group showed better progress than the 1 Hz group in auditory comprehension indexes.

In comparison, the 1 Hz group showed better improvement than the 0.5 Hz group in spontaneous speech indexes. The results of Wang et al. suggest that 0.5 Hz and 1 Hz rTMS can produce respective more advantageous therapeutic effects on different aphasic symptoms, so should different frequencies of rTMS should be selected for targeted treatment to enhance the therapeutic effects of rTMS on PSA patients corresponding to various symptoms of aphasia. More RCTs with different stimulation frequencies of rTMS for PSA need to be conducted in the future to expand the selection of treatment parameters so that we can provide individualized treatment for PSA patients with different symptoms in the clinical treatment of PSA patients.

An expert guideline published in 2021 (74) addresses the safety and recommendations for the use of rTMS in healthy subjects and patient populations. This guideline provides the most up-to-date information on the possible induction of seizures, which are theorized to be the most serious risk of rTMS. It has become apparent that such a risk is low, even in patients taking drugs acting on the central nervous system, at least with the use of traditional stimulation parameters and focal coils for which large data sets are available. However, in this study, we included a total of 819 subjects, but only 4 subjects experienced symptoms such as transient dizziness and nausea, and no patient experienced any seizure symptoms. First, we were not direct participants in this RCT and cannot be certain that these side effects necessarily came from the therapeutic effects of rTMS, and second, if these adverse effects did come as a result of the rTMS intervention, they did not result in any persistent, irreversible changes in the condition of the PSA patients. Finally, compared to the total number of participants in the trial, the number of patients experiencing adverse effects was only 0.5% of the total, making the probability of adverse events extremely low. Considering the clinical application of rTMS, the treatment of PSA patients with rTMS is indeed highly safe.

### 4.1 Study limitations

However, our meta-analysis also has several limitations that should be considered. First, the sample size of our included RCTs was small (*n* < 100), and too small a sample size tends to bias the assessment of treatment effects and overestimate the efficacy of rTMS. Second, although our studies all showed the positive impact of transcranial magnetic stimulation (TMS) in patients with post-stroke aphasia, most did not report patient follow-ups further to confirm the long-term effects of TMS. Third, the studies we included differed regarding the stimulation sites and the number of pulses. The number of available studies did not allow for more detailed subgroup analysis. Fourth, the absence of gray reports may lead to bias in comprehensive analysis results. Meaningful research is more likely to be accepted for publication, making us cautious about jumping to conclusions. Fifth, this study lack of patient-level data and possible cultural/geographical biases (majority of studies from China). Therefore, more multicenter follow-up, double-blind RCTs should be conducted to facilitate longitudinal and cross-sectional comparisons of different stimulation parameters of rTMS, to determine the optimal treatment protocol, and to improve the clinical efficacy of rTMS in patients with PSA. Moreover, heterogeneity in the site of stimulation across studies limits the generalizability of pooled results. Most studies targeted contralesional areas, but some used ipsilesional or bilateral protocols, which may lead to distinct therapeutic trajectories. The absence of detailed subgroup analyses also prevents us from assessing whether different aphasia phenotypes (e.g., Broca, global, motor) respond differently to rTMS. This remains an important future direction for tailoring interventions.

## 5 Conclusion

This study shows that rTMS can safely and effectively improve speech function in patients with post-stroke aphasia (PSA), particularly in auditory comprehension, naming, repetition, and spontaneous speech, which aligns with the findings of Gholami et al. (20) Transient adverse events such as headache, nausea, and dizziness were observed during treatment, but the incidence was very low (0.49%) and the symptoms resolved within 24 h. Furthermore, our systematic review and analysis indicate that different rTMS frequencies produce distinct therapeutic benefits for specific aphasic symptoms: 0.5 Hz rTMS outperforms 1 Hz rTMS in improving auditory comprehension, whereas 1 Hz rTMS is more advantageous for enhancing spontaneous speech. Future multicenter, large-sample randomized controlled trials using different rTMS frequencies are needed to determine the optimal stimulation parameters for PSA.

## Data Availability

The original contributions presented in the study are included in the article/Supplementary material, further inquiries can be directed to the corresponding author.
