# Supplementary figures and images for: Efficacy and safety of repetitive transcranial magnetic therapy for post-stroke aphasia: a systematic review and meta-analysis of randomized controlled trials
Source: Front Neurol. 2025 Oct 20;16:1614586. doi: 10.3389/fneur.2025.1614586 (PMC12580143; doi:10.3389/fneur.2025.1614586)

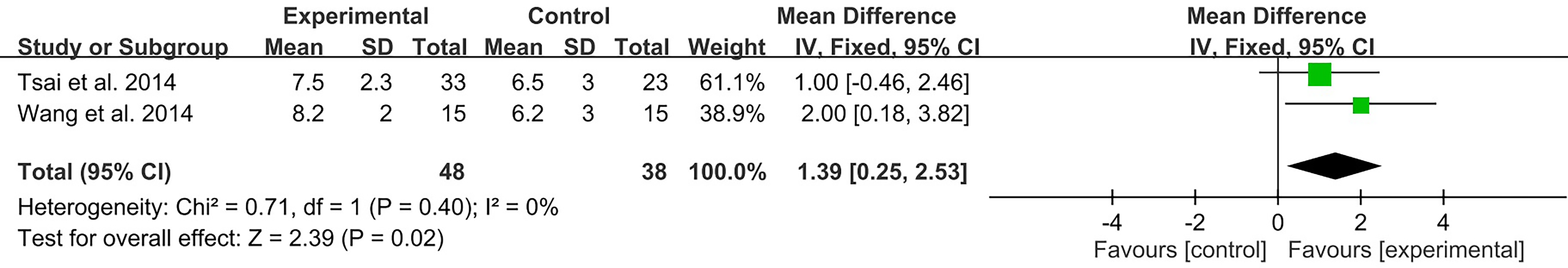

Supplement: Supplementary file 1 [file Image_1.TIF]

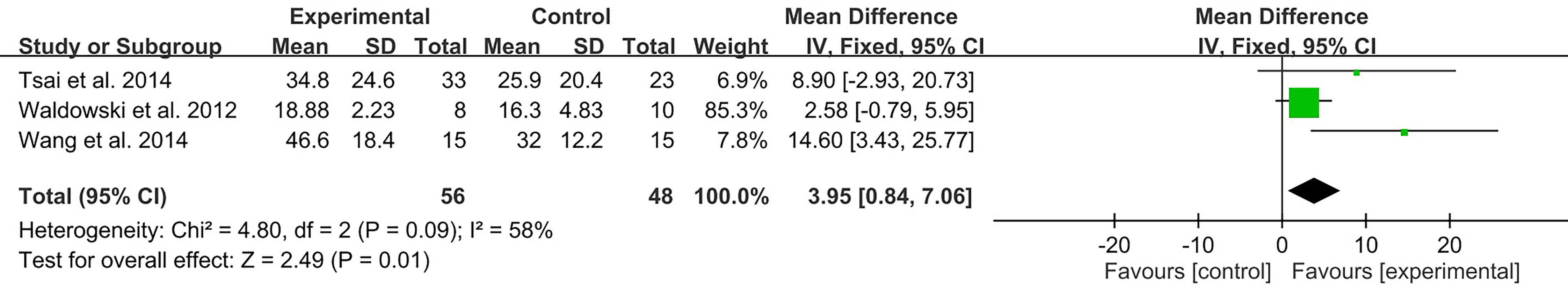

Supplement: Supplementary file 2 [file Image_2.TIF]
